# Imaging neurodegeneration in Down syndrome: brain templates for amyloid burden and tissue segmentation

**DOI:** 10.1007/s11682-018-9888-y

**Published:** 2018-05-11

**Authors:** Patrick J. Lao, Ben L. Handen, Tobey J. Betthauser, Karly A. Cody, Annie D. Cohen, Dana L. Tudorascu, Charles K. Stone, Julie C. Price, Sterling C. Johnson, William E. Klunk, Bradley T. Christian

**Affiliations:** 1 Department of Medical Physics, University of Wisconsin-Madison, 1111 Highland Ave, Madison, WI 53705 USA; 2 Waisman Center, University of Wisconsin-Madison, 1500 Highland Ave, Madison, WI 53705 USA; 3 Department of Psychiatry, University of Pittsburgh, 3811 O’Hara Street, Pittsburgh, PA 15213 USA; 4 Department of Pediatrics, University of Pittsburgh, 4401 Penn Avenue, Pittsburgh, PA 15224 USA; 5 Department of Psychology, University of Pittsburgh, 201 South Bouquet Street, Pittsburgh, PA 15260 USA; 6 Department of Instruction and Learning, University of Pittsburgh, 230 South Bouquet Street, Pittsburgh, PA 15260 USA; 7 Department of Internal Medicine, University of Pittsburgh, 3459 Fifth Avenue, Pittsburgh, PA 15213 USA; 8 Department of Biostatistics, University of Pittsburgh, 130 De Soto Street, Pittsburgh, PA 15261 USA; 9 Department of Cardiovascular Medicine, University of Wisconsin-Madison, 1 South Park Street, Madison, WI 53715 USA; 10 Department of Radiology, University of Pittsburgh, 3600 Forbes @ Meyran Avenues, Pittsburgh, PA 15213 USA; 11 Department of Radiology, Massachusetts General Hospital, 149 13th Street, Charlestown, MA 02129 USA; 12 Department of Medicine-Geriatrics, University of Wisconsin-Madison, 1685 Highland Ave, Madison, WI 53705 USA; 13 Department of Neurology, University of Pittsburgh, 3471 Fifth Avenue, Pittsburgh, PA 15213 USA; 14 Department of Psychiatry, University of Wisconsin-Madison, 6001 Research Park Blvd, Madison, WI 53719 USA

**Keywords:** Down syndrome, Brain template, Alzheimer’s disease, PET, MRI

## Abstract

The focus of Alzheimer’s disease (AD) neuroimaging research has shifted towards an investigation of the earliest stages of AD pathogenesis, which manifests in every young adult with Down syndrome (DS; trisomy 21) resulting from a deterministic genetic predisposition to amyloid precursor protein overproduction. Due to morphological differences in brain structure in the DS population, special consideration must be given to processing pipelines and the use of normative atlases developed for the non-DS population. Further, the use of typical MRI to MRI template spatial normalization is less desirable in this cohort due to a greater presence of motion artefacts in MRI images. The diffuse nature of PiB uptake and comparatively lower spatial resolution of the PET image permits the purposing of this modality as a template for spatial normalization, which can substantially improve the robustness of this procedure in the cases of MRI images with motion. The aim of this work was to establish standardized methods for spatial normalization and tissue type segmentation using DS specific templates in order to perform voxel-wise analyses. A total of 72 adults with DS underwent [^11^C]PiB PET to assess brain amyloid burden and volumetric MRI imaging. A DS specific PiB template for spatial normalization and a set of DS specific prior probability templates were created with two-pass methods. With implementation of this DS specific PiB template, no participants were excluded due to poor spatial normalization, thus maximizing the sample size for PiB analyses in standardized space. In addition, difference images between prior probability templates created from the general population and the DS population reflected known morphological differences, particularly in the frontal cortex. In conclusion, DS specific templates that account for unique challenges improve spatial normalization and tissue type segmentation, and provide a framework for reliable voxel-wise analysis of AD biomarkers in this atypical population.

**Electronic supplementary material:**

The online version of this article (10.1007/s11682-018-9888-y) contains supplementary material, which is available to authorized users.

## Introduction

Trisomy 21 (Down syndrome; DS) is the most common genetic intellectual disability (12-15% of all learning disabilities; Bittles and Glasson 2004) with 1 in every 733 live births in the United States having DS (Centers for Disease Control and Prevention 2006). This represents a 31.1% increase in prevalence from 1979 to 2003 (Shin et al. 2009) mostly due to women conceiving at a later age (Penrose 1954). DS affects brain development, leading to lower brain weight with a small cerebellum, frontal cortex, and temporal cortex, as well as simplified appearances of sulci and a narrow superior temporal gyrus at autopsy (Coyle et al. 1986; Wisniewski 1990). Consistent with autopsy reports, high resolution MRI studies demonstrate lower overall brain volume, as well as a lower volume in the cerebellum, cingulate gyrus, frontal cortex, superior temporal cortex, and hippocampus, compared to the general population (Pinter et al. 2001; Weis et al. 1991). Volumes of subcortical structures, such as the basal ganglia, are preserved in DS (Aylward et al. 1997).

**Fig. 1:**
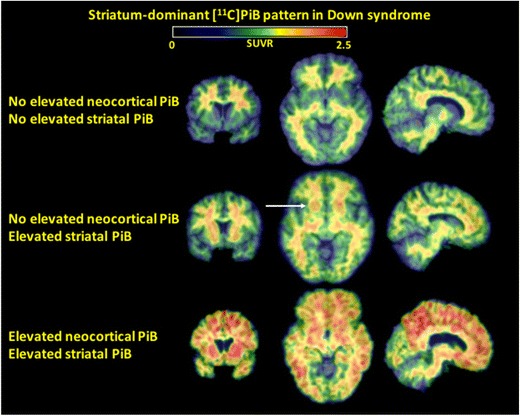
Three representative [^11^C]PiB standard uptake value ratio (SUVR) images. Each row is a representative subject, with the middle row demonstrating the striatum-dominant patterns

The triplicate copy of chromosome 21 leads to the overproduction of amyloid precursor protein (APP; Wiseman et al. 2015), which is thought to contribute to early amyloid plaque accumulation (e.g., beginning in their teens and becoming nearly ubiquitous by 40 years of age; Mann 1988) that manifests with a striatum-dominant (i.e., striatum-first) pattern (Fig. 1; Handen et al. 2012; Lao et al. 2016; Annus et al. 2016). The striatum-dominant pattern of amyloid accumulation in DS differs from the neocortex-dominant pattern in sporadic Alzheimer’s disease (AD), but accumulates at a similar rate in DS as in sporadic AD when stratified by amyloid positivity (Lao et al. 2017a, b; Villemagne et al. 2013). Interestingly, the striatum-dominant pattern also manifests in autosomal dominant AD (ADAD) in which there is a similar genetically deterministic overproduction of amyloid (Villegmagne et al. 2009). AD studies in ADAD and DS have shown striking similarities between themselves, and with sporadic AD, particularly in their overall time course and patterns of neurodegeneration (e.g., glucose metabolism, brain atrophy; Lao et al. 2017a, b; Villemagne et al. 2013; Bateman et al. 2012; Benzinger et al. 2013). Moreover, there is a higher prevalence of AD in DS (up to 75% by 65 years), compared to the general population or other intellectual disabilities (Zigman et al. 1997). The average life expectancy in DS has dramatically increased from 9 to 12 years in 1929-1949 to 55-60 years in 1991-2002 (Penrose 1933, 1954; Bittles and Glasson 2004), making the higher prevalence of AD at middle to late life a greater concern for individuals with DS.

Analysis of brain morphology or pathophysiology often requires spatial normalization and spatial smoothing for voxel-wise statistical testing. Spatial normalization involves an affine transformation to approximately align an image to a template, and a non-linear transformation to ensure that each voxel corresponds to the same anatomical location across subjects (Ashburner et al. 1997; Ashburner and Friston 2000). Two-pass methods are often used in neuroimaging analyses of atypical populations like DS, to create group specific templates that minimize error due to morphological differences (Teipel et al. 2004; Travers et al. 2015; Whitwell et al. 2007). Scans are spatially normalized to the young, healthy control template, and then averaged and smoothed into a group specific template in standardized space. The original scans are spatially normalized a second, independent time to the newly created group specific template.

Similarly, this two-pass method can be used with the DS population to create group specific prior probability templates for tissue type segmentation of MRI images into gray matter (GM), white matter (WM), or cerebrospinal fluid (CSF). Tissue type segmentation involves bias correction, intensity histogram thresholding, and prior probability information about the likelihood of a voxel in standardized space being a particular tissue type based on its location in the brain (Ashburner and Friston 1997). Tissue type segmentation is a common technique used to provide insight into the AD-associated brain atrophy that can occur even prior to disease onset (Dore et al. 2013; Petersen et al. 1999; Chetelat et al. 2002; Fan et al. 2008). However, care must be taken to address the already affected neural substrate in the DS population (White et al. 2003).

The aim of this work was to explore the various methods of spatial normalization and tissue type segmentation that are necessary for voxel-wise analysis of PET and MRI data in the DS population to assess AD associated changes such as amyloid accumulation or gray matter atrophy. Standardized analysis techniques should account for special considerations in imaging the DS population, such as implementing protocols to minimize participant discomfort. DS specific templates for spatial normalization and tissue type segmentation should also account for the unique DS brain morphology, allowing for more precise interrogation of various AD biomarkers. Standardized analysis techniques for DS imaging data would enable results to be directly compared between studies and facilitate faster progression in the field, especially in studies with small DS cohorts.

## Methods

### Down syndrome cohort

Seventy-two participants (38 ± 7 yrs., 38 M/34F) had a baseline scan in a National Institute of Health funded, longitudinal study performed at two imaging facilities (40 at University of Wisconsin-Madison (UW-Madison), 32 at University of Pittsburgh-Medical Center (UPMC)). All participants had genetically confirmed trisomy 21. At entry, all participants were classified as non-demented (i.e., asymptomatic) based on the Dementia Scale for Down Syndrome (DSDS; cognitive cutoff score (CCS) of less than 3), a 60-item measure with favorable specificity and sensitivity (Gedye 1995). Participants were genotyped for the apolipoprotein ε4 allele (APOE4), where APOE4 is the largest, non-deterministic genetic risk factor for AD. Two participants did not have APOE4 information and two other participants had issues with imaging (one did not complete the T1 MRI scan and one did not complete the PET scan). Therefore, 68 participants (38 ± 7 yrs., 37 M/31F) were considered in the generation of DS specific templates.

### [^11^C]PIB PET imaging

[^11^C]PiB is a derivative of a Thioflavin T histological dye for amyloid plaques (Klunk et al. 2003) that has good brain penetrance and specifically binds to fibrillar amyloid plaques. Up to 15 mCi of [^11^C]PiB (>2 mCi/nmol) was delivered intravenously via bolus injection (20-30s). PET data were acquired on Siemens ECAT HR+ scanners at both sites. A 6-10 min ^68^Ge/^68^Ga transmission scan was acquired for attenuation correction of annihilation radiation. PET data were reconstructed with a filtered back-projection algorithm (Direct Inverse Fourier Transform; DIFT) with sinogram trimming to a voxel size of 2.57 mm × 2.57 mm × 2.43 mm and matrix dimension of 128 × 128 × 63 with corrections for detector deadtime, scanner normalization, photon scatter, and radioactive decay.

PET scans were reoriented along the anterior commissure-posterior commissure (AC-PC) line, and inter-frame motion was corrected (AIR version 3.0; Woods et al. 1998). Standard uptake value ratio (SUVR) images were calculated from data 50-70 min post-injection (McNamee et al. 2009) with a cerebellar GM reference region drawn in native space (Klunk et al. 2004; Lopresti et al. 2005; Price et al. 2005).

### Volumetric MRI imaging

T1 weighted 3.0 T MRI scans were acquired on a GE SIGNA 750 (UW-Madison) or a Siemens Magnetom Trio (UPMC). The SIGNA 750 acquired data using a high resolution volumetric spoiled gradient sequence (TI/TE/TR = 450/3.2/8.2 ms, flip angle = 12°, slice thickness = 1 mm no gap, matrix size = 256x256x156). The Magnetom Trio acquired data using a magnetization prepared rapid acquisition gradient echo sequence (MPRAGE; TI/TE/TR = 900/2.98/2300 ms, flip angle = 9°, slice thickness = 1.2 mm, matrix size = 160x240x256). In the 68 available baseline T1 MRI images, 8 (12%) were flagged at UPMC during preprocessing as containing “severe motion” or “significant motion”, leaving 60 T1 MRIs suitable for tissue type segmentation.

T2 weighted MRIs were also acquired on a 3.0 T GE SIGNA 750 (TE/TR = 85/9000 ms, slice thickness = 2 mm no gap, matrix size = 256x256x96) at UW-Madison. T2 weighted MRIs acquired at UPMC were not available for this analysis. Of the 40 available baseline T2 MRI images, 3 (7.5%) were flagged at UPMC during preprocessing as containing “severe motion” or “significant motion”, leaving 37 T2 MRIs suitable for tissue type segmentation.

### Down syndrome specific spatial normalization template

In the atypical DS population, spatial normalization was performed with a two-pass method. PiB SUVR images were coregistered to T1 MRIs in native space. In the first pass, native space T1 MRIs were spatially normalized to the T1 MRI template in standardized space (Montreal Neurological Institute (MNI) space; MNI152 T1 MRI template averaged across 152 subjects) provided in the Statistical Parametric Mapping toolbox (SPM; MATLAB; Ashburner et al. 1997; Ashburner and Friston 2000). The transformation matrices were applied to coregistered, native space PiB SUVR images. The first-pass images in standardized space were visually inspected and qualitatively assessed on cortical outline and striatal placement. A subset of the PiB SUVR images in standardized space were selected from first pass spatial normalizations that were not affected by MRI motion (*n* = 60; see Section 3.1 for more details). The subset was averaged and smoothed (8 mm Gaussian) to create a DS specific PiB template in standardized space (http://www.waisman.wisc.edu/amyloid/Down-Syndrome-Brain-Template.html). The smoothing step compensated for the small subset used to create the template, compared to the MNI152 T1 MRI template. In the second pass, native space PiB SUVR images were spatially normalized to the DS specific PiB template. The transformation matrices were applied to co-registered, native space T1 MRIs. Again, the second-pass images in standardized space were visually inspected and qualitatively assessed on cortical outline and striatal placement.

### Down syndrome specific tissue type segmentation templates

GM volume, determined by tissue type segmentation, is often used as a metric to quantify atrophy and is associated with neuron density (Bourgeat et al. 2010; Chetelat et al. 2010). Tissue type segmentation was similarly performed with a two-pass method. In the first pass, T1 MRIs were segmented in standardized space using GM, WM, and CSF prior probability templates provided in SPM (Ashburner and Friston 1997). A subset of segmented images (*n* = 60) were averaged and smoothed (8 mm Gaussian) to create DS specific GM, WM, and CSF prior probability templates. In the second pass, T1 MRIs were segmented in standardized space using the DS specific prior probability templates. The segmented images were visually inspected for reasonable tissue type classification. Final segmented images had voxel values ranging from 0 to 1, representing the likelihood of each voxel belonging to a specific tissue type.

The segmentation algorithm can incorporate information from more than one MRI per subject to improve tissue type segmentation accuracy. For instance, segmentation can be performed with T1 and T2 MRIs (i.e., multispectral segmentation), rather than just a T1 MRI (i.e., single channel segmentation). Multispectral tissue type segmentation was included to overcome the issue of low quality T1 MRIs for some subjects by including information from a second set of MRIs (e.g., T2 MRIs). T2 MRIs were not available from subjects scanned at UPMC, and, therefore, DS specific multispectral prior probability templates were created from a different subset of scans (*n* = 37) compared to the DS specific single channel (n = 60) prior probability templates.

### Validation of spatial normalization template

To validate use of the DS-specific PiB template, six investigated regions of interest (ROIs; anterior cingulate, frontal cortex, partietal cortex, precuneus, striatum, temporal cortex) as well as a global ROI (cortical mean across the investigated ROIs), were defined using two different methods. On a subset (*n* = 68) of DS PiB SUVR images, hand-drawn ROIs were defined in native space with a set of rules detailing the spatial extent of each region (Supplemental Methods). Additionally, with the same subset, hand-drawn ROIs were created in ITK-SNAP version 3.4.0 on normalized T1 MRIs and combined into a single mask for each ROI (to ensure a proper fit despite any differences that may have persisted after spatial normalization.) These standardized space ROI masks were closely inspected for each region on each subject. To assess if there were any differences between the mean SUVR computed using two different methods, a repeated measures analysis with a fixed method factor and a random subject effect (to account for within subject correlation) was used. The reference method for comparison was the hand drawn ROI’s in native space. The estimated mean differences along with the corresponding 95% confidence intervals (CIs) were calculated. Intra-class correlation coefficients (ICCs) were calculated using a one-way random effects model to assess agreement of the native space and standardized space methods.

## Results

### Standardized space

**Fig. 2:**
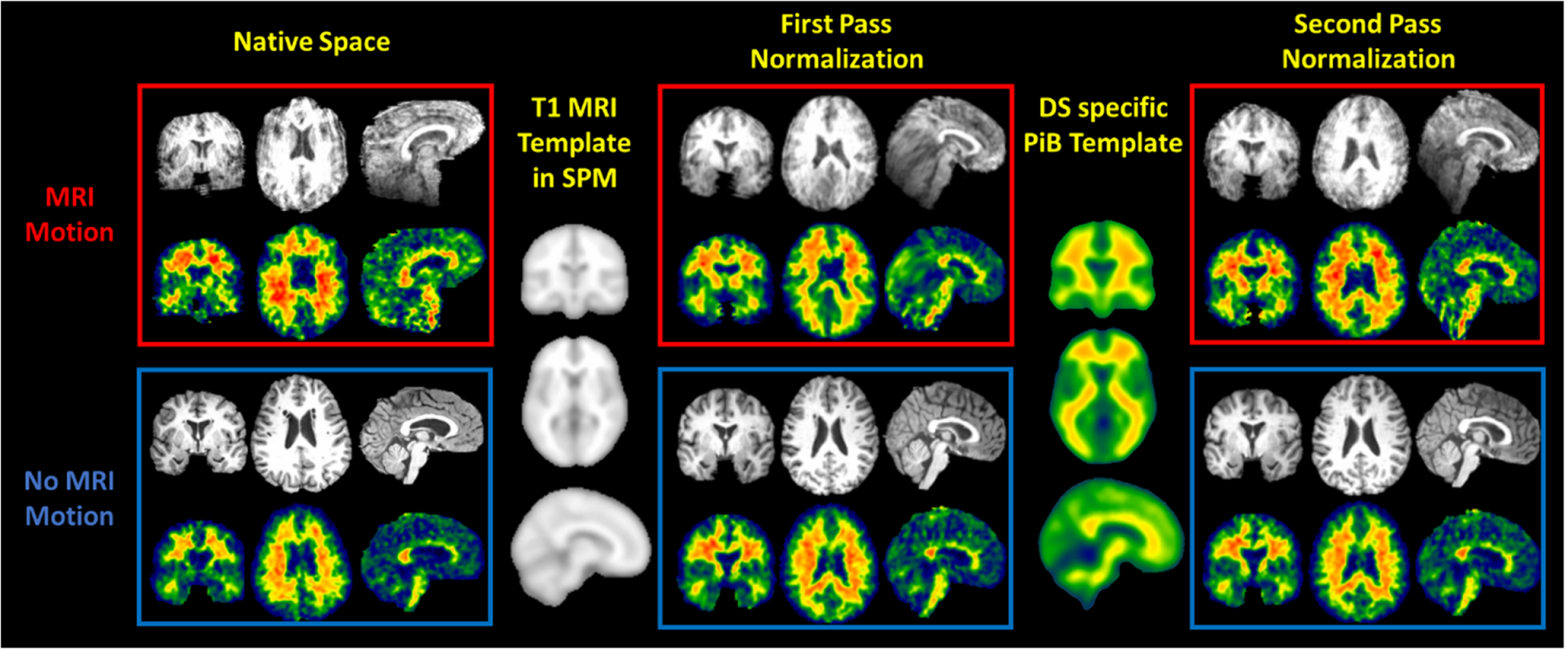
Visual comparison of representative scans with and without MRI motion in native space (red and blue boxes, respectively), and in standardized space after first pass normalization (Native space MRI to T1 MRI template (MNI152)) or second pass normalization (Native space PiB SUVR to DS specific PiB template)

In the 68 available baseline T1 MRI images, 8 (12%) were flagged during preprocessing as containing “severe motion” or “significant motion”. In Fig. 2, the first and second pass normalizations are shown for two representative scans. The first representative scan (Fig. 2, red box) illustrates poor native space T1 MRI quality and orientation. After first pass normalization, the posterior brain was highly distorted, producing a “smearing” effect that was transferred to the co-registered PiB SUVR image. A portion of the frontal cortex was outside the field of view in native space, but was over-warped by extensively increasing the curvature. The corpus callosum was also visibly distorted. The second representative scan (Fig. 2, blue box) illustrates suitable native space T1 MRI quality and orientation, and did not demonstrate problems after the first pass spatial normalization. Therefore, a subset of PiB SUVR images, represented by the second scan (Fig. 2, blue box), were used to create the DS specific PiB template.

After the second pass normalization, the first representative scan (Fig. 2, red box) demonstrated less smearing in the posterior brain, and the frontal cortex and corpus callosum were no longer visibly distorted. Importantly, in the second representative scan (Fig. 2, blue box), the result from the second pass normalization compared to that from the first pass normalization were not visually different. Quantitatively, the percent difference in mean PiB SUVR between the first and second pass normalization was minimal (−0.88 ± 1.97%; Lao et al. 2016) in the 60 subjects without motion. Importantly, no subjects were excluded from PiB SUVR analysis in standardized space due to poor spatial normalization using the DS specific PiB template (*n* = 68; compared to *n* = 60 using MNI152 T1 MRI template).

### Tissue type segmentation

**Fig. 3:**
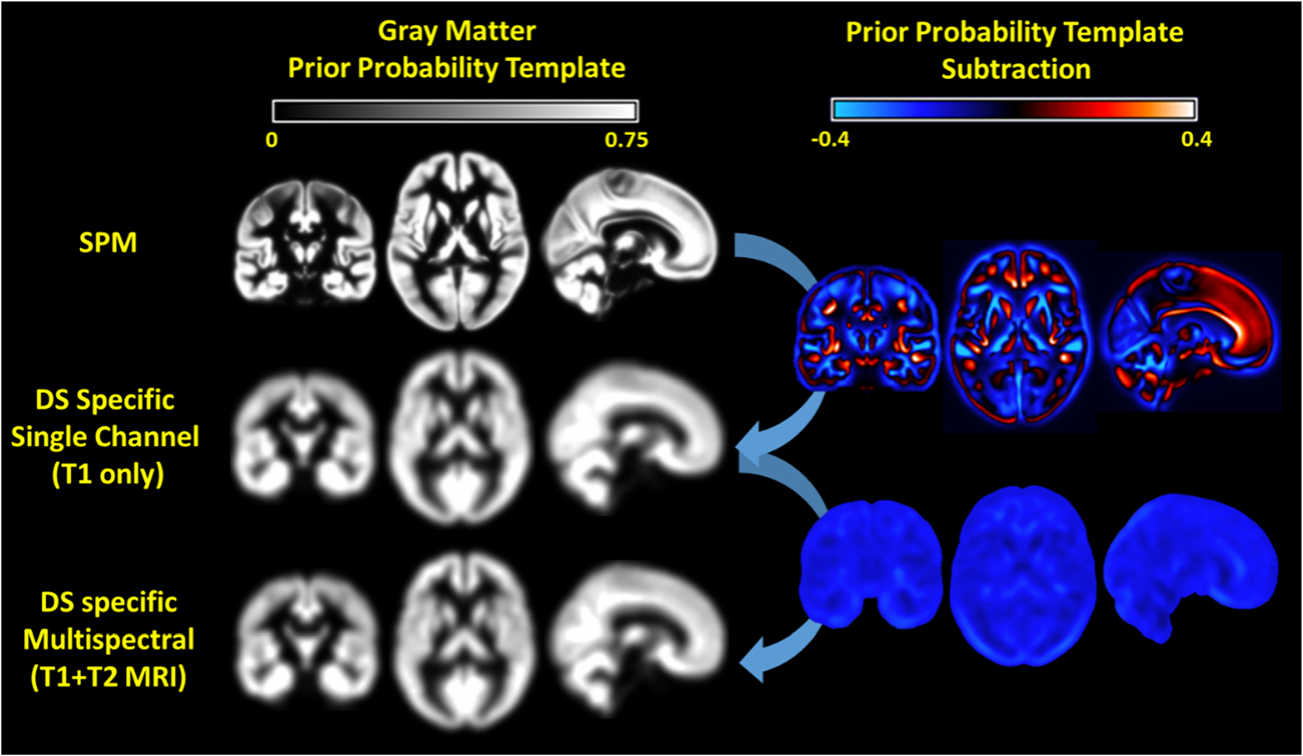
Gray matter prior probability templates (SPM, DS specific single channel, DS specific multispectral), and the subtraction images between them (SPM – DS specific single channel; DS specific single channel – DS specific multispectral)

Figure 3 shows the young, healthy GM prior probability template provided in SPM, as well as the DS specific single channel and multispectral GM prior probability templates. The subtraction image between the SPM and DS specific single channel GM prior probability templates had a blue halo (i.e., negative values in which SPM template < DS specific template) surrounding the whole brain, as well as GM regions (i.e., in WM; Fig. 3). These negative values were a consequence of the smoothed DS specific template extending farther spatially than the non-smoothed SPM template (i.e., spilling out of GM regions). Importantly, there were areas of bright red (i.e., large positive values in which SPM template >> DS specific template) in the frontal cortex, and red (i.e., positive values in which SPM template > DS specific template) in the temporal cortex and cerebellum. The areas of darker red (i.e., small positive values in which SPM template > DS specific template) could reflect generally lower GM volumes in DS compared to the general population, or another consequence of smoothing where values are being pulled down in addition to being spatially broadened. Visually, there are slight intensity differences between the single channel and multispectral templates, but the subtraction image showed only slightly blue regions (i.e., small negative values in which single channel < multispectral) with values less than −0.10, representing changes smaller than 10%.

### Region of interest method validation

Table 1 shows mean SUVRs and standard deviations from native space and standardized space ROIs. Table 2 shows the results from the repeated measures model represented as the mean estimated difference and 95% CI using native space values as the reference method. With the exception of the precuneus, the SUVR values were generally higher for ROIs in native space compared to ROIs in standardized space; however, the extent of the difference varied by ROI. Table 3 shows the results of ICCs comparing agreement of standardized space SUVRs with native space SUVRs. ICCs were interpreted as strong agreement between the two methods when above 0.7, and as near perfect agreement when above 0.8. All standardized space derived SUVRs and native space derived SUVRs exceeded strong agreement, although the extent of the agreement varied by ROI.

**Table 1:** Descriptive Statistics for computed SUVR’s for each ROI/method

ROI	Methods	
	Hand Drawn in Native Space (n=70)	Hand Drawn in Template Space (n=70)
Anterior Cingulate	1.36 (0.28)	1.30 (0.29)
Frontal Cortex	1.26 (0.28)	1.21 (0.18)
Parietal Cortex	1.28 (0.23)	1.10 (0.21)
Precuneus	1.37 (0.29)	1.44 (0.29)
Striatum	1.46 (0.52)	1.39 (0.45)
Temporal Cortex	1.28 (0.22)	1.20 (0.15)
Global	1.33 (0.29)	1.27 (0.25)

Values are presented as means and standard deviations for each ROI SUVR

**Table 2:** Repeated measures model: Mean estimated differences and 95% CI

ROI	Native Space vs Template Space	Sig. (2-tailed) *p*
Anterior Cingulate	0.050 (0.039, 0.062)	<.001
Frontal Cortex	0.055 (0.017, 0.092)	.006
Parietal Cortex	0.187 (0.161, 0.212)	<.001
Precuneus	-0.070 (-0.083, -0.057)	<.001
Temporal Cortex	0.082 (0.057, 0.108)	<.001
Striatum	0.056 (0.031, 0.082)	<.001
Global	0.061 (0.047, 0.075)	<.001

Results are presented as mean SUVR estimated differences between the Hand Drawn Native Space method and the Hand Drawn Template Space method and their 95% CI

**Table 3:** Intra-class correlation coefficients (ICC’s) were calculated using one-way random effects model. The ICC’s and their 95% confidence intervals were computed between the Native Space ROI’s and the Template Space ROI’s

ROI	ICC CI (Lower Bound, Upper Bound)	Significance *p*
Anterior Cingulate	0.984 (0.975, 0.990)	<.001
Frontal Cortex	0.855 (0.766, 0.910)	<.001
Parietal Cortex	0.738 (0.579, 0.837)	<.001
Precuneus	0.977 (0.963, 0.986)	<.001
Striatum	0.983 (0.973, 0.990)	<.001
Temporal Cortex	0.853 (0.764, 0.909)	<.001
Global	0.973 (0.957, 0.983)	<.001

## Discussion

Genetically deterministic amyloid accumulation in DS represents a unique opportunity to investigate early AD associated changes without having to predict which young, healthy, cognitively stable individuals will eventually develop abnormal AD biomarkers because nearly all adults with DS demonstrate amyloid deposition by age 40. However, there are special considerations for imaging studies in any atypical population because templates for spatial normalization or tissue type segmentation are created from large samples of healthy individuals. To be consistent with much of the neuroimaging field, a DS specific T1 MRI template would be utilized. However, for this population, consistent MRI quality was more problematic than PET quality. The high-resolution MRI scans were more sensitive to subject motion than the lower resolution PET scans. Participant comfort likely played a role, as subject motion tended to be more exascerbated in the noisy and confined environment of the MRI scanner than the silent and open environment of the PET scanner.

In addition to fewer motion artefacts in PET imaging of this DS population, the diffuse nature of PiB uptake and comparatively lower spatial resolution of the PET image permits the purposing of this modality as a template for spatial normalization. Moreover, the measurable WM uptake of [^11^C]PiB (Fodero-Tavoletti et al. 2009) and large proportion of scans with low PiB SUVR in cortical and subcortical regions made the DS specific PiB template visually similar to a T1 MRI template. Importantly, spatial normalization appeared acceptable even for subjects with high PiB SUVR. High PiB SUVR appears diffusely throughout cortical and subcortical regions, as opposed to appearing in scattered, focal spots, and the spatial normalization algorithm simply uses high contrast regions as landmarks without differentiating if WM or GM has higher SUVR.

There are known morphological differences between brains in DS and the general population, and the DS specific prior probability templates reflected lower GM volume in the frontal cortex, temporal cortex, and cerebellum, as well as a generally lower GM volume (Coyle et al. 1986; Wisniewski 1990). Incorportating more information with a multispectral segmentation did not substantially improve the DS specific prior probability templates. In fact, the lack of T2 MRIs from one site combined with the increased risk of motion (i.e., two scans have to be motion-free compared to just one scan) made multispectral segmentation an unfavorable option.

Mean PiB SUVR in standardized space demonstrated strong agreement with that in native space, indicating minimal change in PiB SUVR after spatial normalization using the DS specific PiB PET template. In addition, normalizing to a PET template allowed for regional SUVR extraction without requiring MRI acquisition. While nearly all ROIs were above an ICC of 0.8, discrepancies between the methods could be due to smoothing or inter-rater reliability of the ROI drawing methods. In addition, these results incorporate subject bias by implementing the DS specific template for ROI analyses on the same subjects used to create the template.

The main limitation of this study is the lack of a standardized method for analyzing imaging processing pipelines. A bias measurement in the form of a difference image between native space PiB SUVR and standardized space PiB SUVR cannot be performed since the images are in different spaces. An exploration of the effect of spatial normalization using the MNI152 T1 MRI template and the DS specific PiB template has been shown to be minimal for scans without motion (mean percent difference < 1%; Lao et al. 2016). Similarly, a bias measurement in the form of a difference image between native space GM volume and standardized space GM volume cannot be performed since the images are in different spaces. Ongoing work is necessary to explore the effect of population-based templates on mean PiB SUVR. As a result, our method of validation relied on careful visual inspection and comparable ROIs applied across native and standardized spaces.

## Conclusion

A DS specific PiB template for spatial normalization is facilitated by the diffuse nature of amyloid accumulation and is advantageous due to MRI motion in this DS cohort. Spatial normalization is virtually unchanged in scans without MRI motion (<1%), but is substantially better in scans with MRI motion. Importantly, a DS specific PiB template provides an avenue for PET analysis that is independent of MRI acquisition. DS specific prior probability templates reflected the characteristic DS brain morphology, and has the potential to improve the accuracy of GM volume estimation for investigating AD associated brain atrophy. ROIs in PiB SUVR standardized space do not rely on MRI quality and can be used in tandem with voxel-wise analysis. Ultimately, standardization of analyses methods of DS imaging data will allow for an easier interpretation of results across studies, improving the overall understanding of AD biomarkers in the DS population.

## Electronic supplementary material

ESM 1 (DOCX 13 kb)
