# Supplementary material for: Imaging neurodegeneration in Down syndrome: brain templates for amyloid burden and tissue segmentation
Source: Brain Imaging Behav. 2018 May 11;13(2):345–53. doi: 10.1007/s11682-018-9888-y (PMC6230506; doi:10.1007/s11682-018-9888-y)
Supplement: Supplementary file 1 — (DOCX 13 kb) [file 11682_2018_9888_MOESM1_ESM.docx]

SUPPLEMENTAL DATA

Hand-drawn ROI Definition

1. Anterior cingulate gyrus: 5 planes above and 5 planes below genus of corpus callosum; anterior boundary: frontal cortex; posterior boundary: corpus callosum; lateral boundary: anterior cingulate sulcus.
2. Frontal cortex: 5 planes above and 5 planes below genus of corpus callosum; anterior boundary: front of brain; posterior lateral boundary: Sylvian fissure and circular sulcus; posterior medial boundary: anterior cingulate.
3. Parietal cortex: 5 planes above occipital cortex; anterior boundary: superior temporal sulcus (inferiorly) and posterior branches of post-central sulcus (superiorly); posterior medial boundary: intraparietal sulcus.
4. Precuneus: 5 planes above and 10 planes below the most superior point of the parieto-occipital sulcus; anterior boundary: posterior branch of cingulate sulcus (superiorly) and corpus callosum (inferiorly); posterior boundary: back of brain (superiorly) and parieto-occiptal sulcus (inferiorly).
5. Anterior ventral striatum: 5 planes above most ventral point of caudate; anterior boundary: caudate; posterior boundary: half of putamen; include internal capsule.
6. Lateral temporal cortex: 5 planes below top of middle temporal cortex; anterior boundary: superior temporal sulcus; posterior boundary: temporo-occipital sulcus.
7. Global: Non-weighted average of previous 6 ROIs.
